# PARP1 expression, activity and *ex vivo* sensitivity to the PARP inhibitor, talazoparib (BMN 673), in chronic lymphocytic leukaemia

**DOI:** 10.18632/oncotarget.6287

**Published:** 2015-11-02

**Authors:** Ashleigh Herriott, Susan J. Tudhope, Gesa Junge, Natalie Rodrigues, Miranda J. Patterson, Laura Woodhouse, John Lunec, Jill E. Hunter, Evan A. Mulligan, Michael Cole, Lisa M. Allinson, Jonathan P. Wallis, Scott Marshall, Evelyn Wang, Nicola J. Curtin, Elaine Willmore

**Affiliations:** ^1^ Newcastle Cancer Centre at the Northern Institute for Cancer Research, Medical School, Newcastle University, Newcastle-upon-Tyne, UK; ^2^ Laboratory of Lymphocyte Signaling and Oncoproteome, University Hospital of Cologne, Cologne, Germany; ^3^ Institute of Cell and Molecular Biosciences, Medical School, Newcastle University, Newcastle-upon-Tyne, UK; ^4^ Institute of Medical and Biological Engineering, University of Leeds, Leeds, UK; ^5^ Department of Haematology, Freeman Hospital, Newcastle upon Tyne, UK; ^6^ Department of Haematology, City Hospitals Sunderland NHS Trust, Sunderland, UK; ^7^ Biomarin Pharmaceutical Inc., Novato, California, USA

**Keywords:** PARP, CLL, talazoparib, DNA repair, ATM

## Abstract

In chronic lymphocytic leukemia (CLL), mutation and loss of p53 and ATM abrogate DNA damage signalling and predict poorer response and shorter survival. We hypothesised that poly (ADP-ribose) polymerase (PARP) activity, which is crucial for repair of DNA breaks induced by oxidative stress or chemotherapy, may be an additional predictive biomarker and a target for therapy with PARP inhibitors.

We measured PARP activity in 109 patient-derived CLL samples, which varied widely (192 – 190052 pmol PAR/10^6^ cells) compared to that seen in healthy volunteer lymphocytes (2451 – 7519 pmol PAR/10^6^ cells). PARP activity was associated with PARP1 protein expression and endogenous PAR levels. PARP activity was not associated with p53 or ATM loss, Binet stage, *IGHV* mutational status or survival, but correlated with Bcl-2 and Rel A (an NF-kB subunit). Levels of 8-hydroxy-2′-deoxyguanosine in DNA (a marker of oxidative damage) were not associated with PAR levels or PARP activity. The potent PARP inhibitor, talazoparib (BMN 673), inhibited CD40L-stimulated proliferation of CLL cells at nM concentrations, independently of Binet stage or p53/ATM function.

PARP activity is highly variable in CLL and correlates with stress-induced proteins. Proliferating CLL cells (including those with p53 or ATM loss) are highly sensitive to the PARP inhibitor talazoparib.

## INTRODUCTION

B-cell chronic lymphocytic leukaemia (CLL) patients have a variable disease but the onset of molecular profiling has revolutionised our understanding of CLL, and identified recurrent mutations and novel targets that may be exploited therapeutically. Current treatment protocols have improved response rates for many patients [1] and newer approaches targeting B-cell receptor (BCR) signalling show great promise in clinical trials [2]. Despite these advances, CLL remains incurable, and since CLL patients are predominantly elderly, they cannot tolerate aggressive therapies. The development of biomarkers to stratify subgroups of patients for personalised medicine is therefore crucial to achieve the best therapeutic outcomes for these patients.

Cytogenetic abnormalities including mutation and loss of p53 and ataxia telangiectasia mutated kinase (ATM) (del(17p) and del(11q) respectively) confer chemoresistance and are associated with poorer response and shorter overall survival [3, 4]. Both ATM and p53 signal DNA double-strand breaks (DSB) with ATM also promoting their high-fidelity repair by homologous recombination repair (HRR). These phenotypes were recently found to impact on the newer kinase inhibitors that target BCR signalling. Although the mechanism of action of the BTK inhibitors is p53-independent, underlying genomic instability in patients with p53 and ATM loss can result in acquired mutations in the kinase, resulting in drug resistance [5].

We reasoned that genomic instability arising from unresolved single-strand DNA breaks may also be important in response to DNA damaging therapy in CLL. Poly(ADP-ribose) polymerase-1 (PARP1) is activated by DNA strand breaks and is pivotal in the signalling to their repair [6]. Since PARP1 has a key role in the response to DNA damage, it is currently being pursued as a drug target in several clinical trials [7]. We co-developed the first clinically-used PARP inhibitor [8] for combination chemotherapy but subsequent studies identified the synthetic lethality of PARP inhibitor monotherapy in HRR-defective (HRD) tumours, notably those with BRCA mutations [9, 10]. Importantly, PARP1 is known to be part of the first line of defence against oxidative stress, which is increased in cancer and associated with poor prognosis in CLL [11].

In agreement with the known synthetic lethality of PARP inhibitors in HRD tumours, ATM-defective CLL can call be selectively targeted by the PARP inhibitor, olaparib [12]. A phase I/II clinical trial is investigating the use of olaparib in CLL patients stratified by ATM status (11q deletion or ATM mutation: ISRCTN34386131 http://www.isrctn.com/ISRCTN34386131) and phase I studies of talazoparib have been undertaken in haematological malignancies (NCT01399840).

A comprehensive analysis of PARP1 function in CLL is lacking. We previously observed that PARP activity was very high in a pilot cohort of CLL cases [13] and hypothesised that defects in DNA repair (e.g. loss of p53 or ATM function), response to oxidative stress or oncogene activation could lead to up-regulation of PARP1 and greater reliance on PARP activity for survival of CLL cells. We hypothesised therefore that PARP activity could not only be a key determinant of patient sensitivity to chemotherapies that stimulate PARP activity, but also to PARP inhibitors themselves. Here, we analysed the expression and activity of PARP1 in a large cohort of patient-derived CLL cells (*n* = 109). We found that PARP activity was highly variable in CLL patients, and tended to be higher than PBMCs from healthy volunteers (HV). PARP activity and/or PARP1 protein levels were associated with levels of the antiapoptotic protein, Bcl2, and the DNA binding of the stress-induced transcription factor NF-kB subunit, RelA. PARP activity was not associated with ATM dysfunction or levels of oxidative DNA damage and the potent PARP inhibitor talazoparib inhibited the growth of patient derived CLL cells at low concentrations, even in cells with functional HRR.

## RESULTS

### PARP activity is more variable in CLL cells than in PBMCs from healthy volunteers

**Figure 1:**
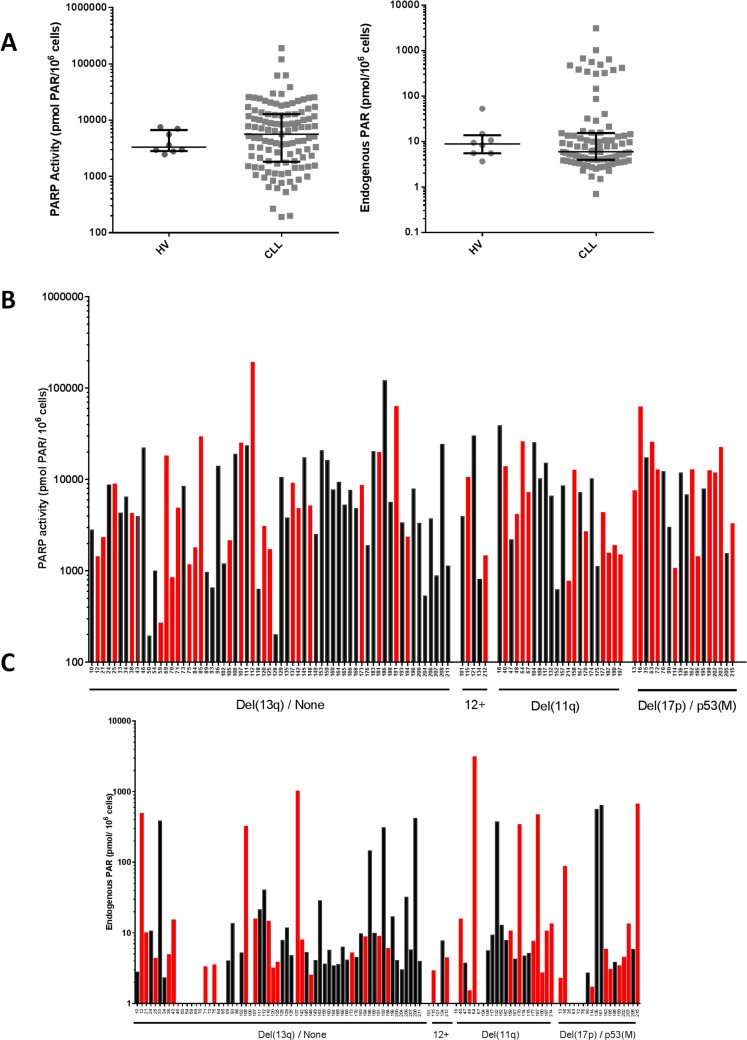
PARP catalytic activity in CLL cells PARP activity was determined by measuring the product (PAR) using a GCLP validated assay (*n* = 109 CLL cases). **A.** PARP activity (left) and endogenous PAR levels (right) in CLL cells and PBMCs from HV. Lines and error bars represent median and 25th/75th centiles. PARP activity **B.** and endogenous PAR levels **C.** in CLL cells is stratified by cytogenetic abnormalities from patients who had received (red bars) or not received (black bars) prior therapy. Samples are classified as either ‘del(13q)/none’ (patients with del(13q) and those who had no cytogenetic abnormalities), ‘12+’ (patients with trisomy 12), ‘del(11q)’, or del(17p)/p53(M) (loss and/or mutation of p53). All data are from duplicate determinations in a single assay.

PARP activity and endogenous PAR levels in patient-derived CLL samples (109 and 90 samples, respectively) were more variable than in PBMCs from HV (*n* = 8, age 25-51, mean 37). In CLL samples there was an approximately 1,000-fold difference from highest to lowest (192- 190052, mean = 11968; median = 5615 pmol PAR formed/10^6^ cells, coefficient of variation = 1.92) compared to PBMCs (3-fold variation) from HV (range = 2452 - 7519, mean = 4365; median = 3315 pmol PAR formed/10^6^ cells, coefficient of variation = 0.47) (Figure 1A left). Similarly, endogenous PAR levels were also highly variable (> 4,000-fold) in CLL cells (0.7 - 3090 pmol, mean = 110; median = 6.0 pmol PAR/10^6^ cells, coefficient of variation = 3.33) compared to HV lymphocytes (3.66 - 52.9 pmol mean = 13.8; median = 8.8 pmol PAR/10^6^ cells, coefficient of variation = 0.88) and there was a population of cells that had particularly high levels (Figure 1A, right). PARP activity significantly correlated with endogenous PAR levels (*p* = 0.01 *r* = 0.27; Supplementary Figure S1). Variable PARP activity and endogenous PAR levels were observed across all cytogenetic groupings with no obvious relationship between PARP activity (Figure 1B) or endogenous PAR levels (Figure 1C) and cytogenetic abnormalities, and no obvious distinction between treated and untreated patients. Some patients' CLL cells were sampled on more than one occasion from diagnosis through their disease course, and measurement of PARP activity did not reveal any particular trend in PARP activity over the course of disease (supplementary Figure S2). These data suggest that neither cytogenetics nor prior therapy influence PARP activity in CLL.

### Cellular PARP activity and Bcl-2 levels are related to PARP1 levels and RelA DNA binding is associated with PARP activity

**Figure 2:**
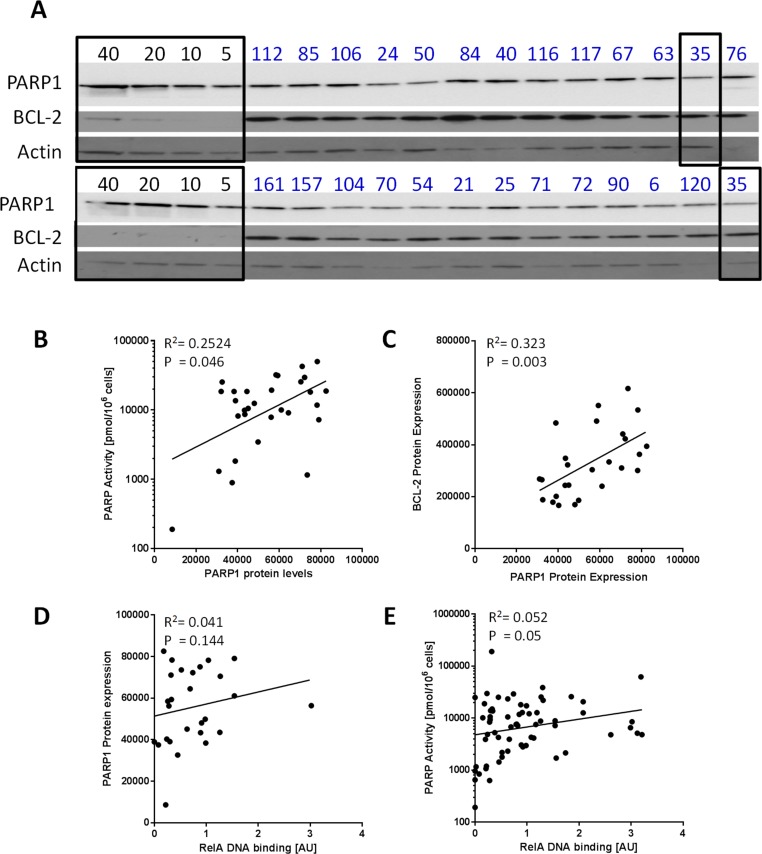
PARP1 protein levels and activity in relation to Bcl2 expression and RelA DNA binding **A.** Whole cell lysates were prepared from CLL cases (*n* = 30) and expression of PARP1 and Bcl2 was measured by Western blot (two blots shown) and quantified using chemiluminescence detection and interpolation from a standard curve generated by probing known concentrations of K562 whole cell lysates (5, 10, 20 and 40 μg, as shown) for PARP1 and Bcl2 (highlighted in black boxes): sample 35 (outlined on right) was evaluated in both blots with similar results. The correlation between PARP1 expression and PARP activity (n = 30) **B.** Bcl2 expression (*n* = 26) **C.** and nuclear NF-κB (RelA) (*n* = 27) **D.** was analysed. RelA binding was also correlated with PARP1 activity (*n* = 65) **E.**. All data are from single determinations.

Since the variation in PARP activity did not correlate with cytogenetic abnormalities we investigated if it was dependent on differential expression of PARP1 and measures of anti-apoptotic and stress signalling. Bcl2 and PARP1 protein expression was analysed by Western blotting (Figure 2A). PARPa aactivity correlated with PARP1 protein levels (*p* = 0.046, Spearman's rank (*p* = 0.138 with Bonferroni correction)) (Figure 2B) although the relationship was not strong (*r* = 0.367). Interestingly, the levels of the anti-apoptotic protein, Bcl2, which is known to play a major role in CLL [14, 15] significantly correlated with PARP1 levels (*p* = 0.003, Spearman's rank (*p* = 0.009 with Bonferroni correction)) and the relationship was stronger (*r* = 0.562) (Figure 2C). PARP has been implicated in the apoptotic process; not only is it cleaved by caspases but PAR formation promotes apoptosis inducing factor release from the mitochondrion and nuclear translocation triggering caspase-independent apoptosis [16]. However, there was no correlation between PARP1 levels and RelA (a component of the stress-activated pro-survival transcriptional activator protein complex NFkB) binding to DNA (Figure 2D). RelA DNA binding did correlate with PARP activity in the CLL samples (Figure 2E, *n* = 65; *p* = 0.045) but the relationship was weak (*r* = 0.25).

### Elevated PARP activity is not related to p53 or ATM function

**Figure 3:**
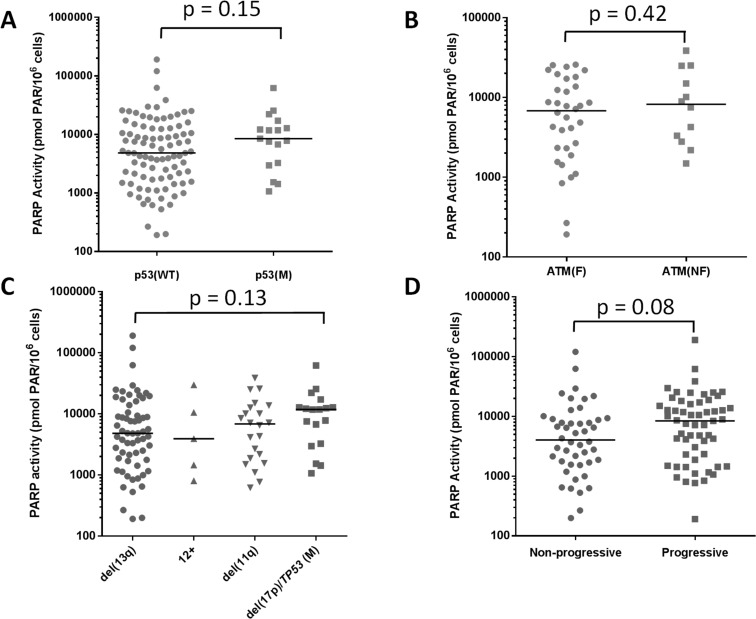
Elevated PARP activity in relation to p53 and ATM function, cytogenetics and disease status PARP activity was examined in relation to p53 function (WT [*n* = 86] or loss or mutation [M, *n* = 16] **A.**, and ATM function (F, ability to auto-phosphorylate (ser1981) and phosphorylate SMC-1, see materials & methods, *n* = 34) or dysfunction (NF *n* = 12) **B.**. Patients were grouped in terms of cytogenetic abnormalities to compare PARP activity **C.**. **D.** PARP activity in relation to disease status: progressive disease (*n* = 46), defined as Binet stage B or C, or non-progressive (*n* = 57), Binet stage A at the time of sample collection. Data are individual sample values (from duplicates in a single assay) with median line shown.

Since the data on cytogenetic abnormalities in Figure 1 may not definitively reflect p53 and ATM function, we analysed the distribution of PARP activity in 102 patients stratified according to their p53 status, including those cases that had *TP53* mutation without del(17p) (*n* = 86 wild type (WT) and p53 mutant (M) *n* = 16) but there was still no significant correlation (*p* = 0.15, Mann Whitney, Figure 3A). Similarly, we examined whether the functional status of ATM, a key player in HRR, had any relationship with PARP activity. Approximately 60% of del(11q) cases have fully functional ATM, and only those cases with biallelic loss of ATM (with mutation on the remaining allele) have non-functional ATM. Therefore, we stratified the del(11q) group according to ATM functional status, i.e. the ability to auto-phosphorylate (ser1981) and phosphorylate SMC-1, (Supplementary figure S3). In the 46 cases evaluated, there was no difference in PARP activity between those with functional (*n* = 34) or non-functional (*n* = 12) ATM (*p* = 0.42) (Figure 3B, supplementary Table S2). Similarly, endogenous PAR levels did not correlate with p53 or ATM function (supplementary Figure S4A, S4B, Table S2). PARP activity was not significantly associated with any particular cytogenetic abnormality (del(13q) *versus* del(17p)/*TP53*, *n* = 98, *p* = 0.13 Mann Whitney; Figure 3C). Similarly, endogenous PAR levels were not associated with cytogenetic abnormalities, supplementary Figure S4C). PARP activity had a tendency to be higher in patients with progressive disease, defined as Binet stage B or C (*n* = 46), compared to those with non-progressive disease, Binet stage A, (*n* = 57) but again, this was not significant (*p* = 0.08 Mann Whitney, Figure 3D). Endogenous PAR levels did not correlate with disease progression (*n* = 84;*p* = 0.47, supplementary Figure S4D). Neither PARP activity nor endogenous PAR levels were related to *IGHV* mutation status (*p* = 0.94 and 0.39, respectively), data not shown.

### PARP activity and endogenous PAR levels are not predictive of survival in CLL

Since PARP activity is a continuous variable, we used ROC curve survival analysis to examine the impact of PARP activity on patient outcome. Maximally stimulated PARP activity and basal PAR levels did not have clinically relevant discriminatory power using ROC curve survival analysis at 2.5 years and 5 years (30 and 60 months) post-diagnosis (supplementary figure S7). AUC values of below 0.75 were deemed to be of no clinical significance [17] with relation to PARP as a prognostic marker in CLL.

### 8-OHdG levels do not correlate with PARP activity, endogenous PAR levels, cytogenetic abnormalities, p53 functional status, or treatment status

**Figure 4:**
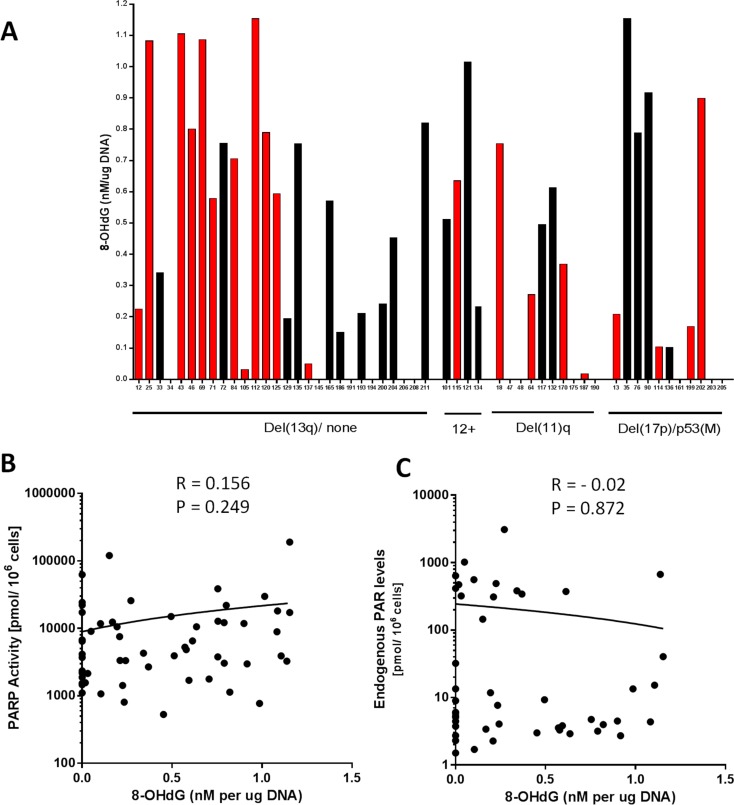
Oxidative DNA damage in relation to cytogenetic abnormalities, PARP activity and endogenous PAR levels 8-hydroxy-2′-deoxyguanosine (8-OHdG) levels, quantified by ELISA in 56 CLL samples in relation to cytogenetic abnormalities and prior therapy (red bars) or untreated (black bars) **A.**. PARP1 activity (B; *n* = 56) and endogenous PAR levels (C; *n* = 47) in relation to 8-OHdG levels. All data are from single determinations.

PARP is activated by DNA breaks, with the most common endogenous cause of these breaks being oxidative stress/reactive oxygen species (ROS). 8-OHdG is the predominant form of ROS-induced DNA damage. ROS levels are reported to be higher in CLL and associated with poor prognosis [11]. Therefore, to determine if ROS-induced DNA damage was associated with cytogenetic abnormalities or PARP activity, or if the endogenous PAR levels reflected the level of ongoing DNA damage, we measured 8-OHdG in DNA extracted from 56 treatment-naïve or pre-treated CLL patient samples (Figure 4). In particular, we wanted to determine if the outlier population of cells with high endogenous PAR (observed in Figure 1A) was due to high levels of oxidative stress. There was no relationship between 8-OHdG levels and the cytogenetic groups or in response to prior therapy (Figure 4A), PARP activity (Figure 4B) or endogenous PAR (Figure 4C), implying that the highly variable PARP activity in CLL does not reflect levels of oxidative stress.

### Talazoparib inhibits the growth of CLL cells irrespective of ATM and HRR status

**Figure 5:**
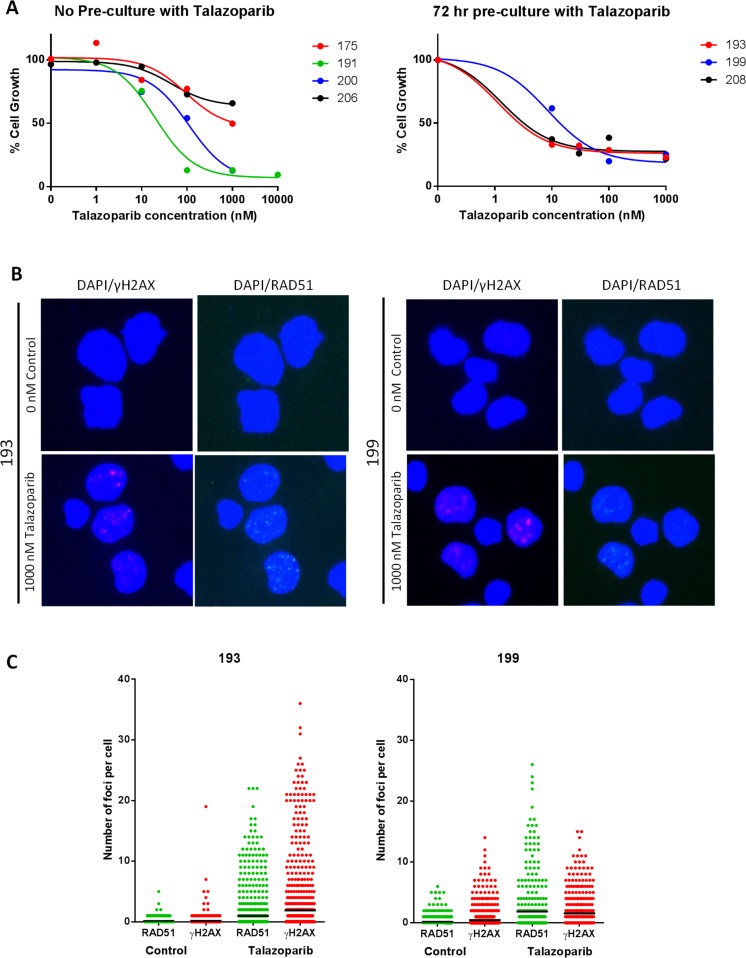
Growth inhibition by potent PARP inhibitor, talazoparib in ATM dysfunctional and HRR functional CLL cells **A.** Concentration response curves for CLL samples stimulated to proliferate on a CD40L feeder layer and treated with 0 – 1000 nM talazoparib. Left graph shows concentration response using method 1, right graph using method 2 (see supplementary Figure S6 for details of drug exposure). Curves are representative of change in cell numbers between 96 - 168 hr exposure. HRR status was determined after 144 hr exposure to 1,000 nM talazoparib by measuring γH2AX immunofluorescence to confirm DNA replication fork collapse, and RAD51 to confirm HRR functionality. Example photomicrographs (x40) are shown in panel B, and data from at least 500 nuclei/treatment (analysed by image J) are shown in panel C (bold lines are median values).

By culturing CLL cells on an irradiated monolayer expressing the CD40 ligand (CD40L), CLL cells can be induced to proliferate, making growth inhibition studies possible [18], and pilot studies confirmed induction of proliferation (Supplementary Figure S5). PARP inhibitors are synthetically lethal to cells with HRR dysfunction and ATM is thought to function in the HRR pathway [12]. In initial studies using CLL samples from 4 patients, (including an ATM dysfunctional and a del11q case) proliferating CLL cells cultured on the CD40L-expressing cells for 72 hr were exposed to talazoparib (0-10,000 nM) (Method 1). Cell counting over 96-168 hr, (i.e. 72 hr exposure) revealed a concentration-dependent suppression of cell growth (Figure 5A left panel) with GI_50_ values between 19 and > 1000 nM talazoparib (Table 1). It has been suggested that high PARP activity is associated with HRR defects [19], although our data do not indicate that ATM defects are associated with higher PARP activity (Figure 3B). To investigate if PARP activity was related to HRR status and hence sensitivity to talazoparib, 3 further CLL samples (193, 199 and 208) with high, medium or low PARP activity and low or high endogenous PAR levels (Table 1) were stimulated to proliferate on a CD40L-expressing layer, and exposed to talazoparib (0 nM - 1,000 nM) after a 72 hr talazoparib pre-exposure period (method 2). Cell counts over the same 96-168 hr time-period demonstrated a profound suppression of growth by all concentrations of talazoparib with GI_50_ values of < 25 - 30 nM (Figure 5A right panel, supplementary figure S6). Although these data seem to imply that ATM dysfunction confers resistance to talazoparib, a direct comparison cannot be made between data from method 1 and 2 due to the differences in the pre-exposure period. However, using method 1, sample 200 (which had non-functional ATM) had a 10-fold higher GI_50_ value than sample 191 (which had functional ATM).

**Table 1:** Summary of patient information for cases used in growth inhibition studies

Patient ID	Cytogenetic Abnormalities	Binet Stage	p53 status	ATM Status	Maximally Stimulated PARP Activity [pmol/>10^6^ cells]	Endogenous PAR levels [pmol/10^6^ cells]	8-OHdG [nM/μg DNA]	talazoparib GI50 [nM]
175	Del(11q)	B	WT	F	1105.1	5.14	0	994.0
191	Del(13q)	A	WT	F	62676.3	8.9	0	19.39
200	Del(13q)	A	WT	NF	3317.3	4.1	0.242	191.2
206	Del(13q)	A	WT	-	3701.0	32.1	0	>1000
193	Del(13q)	-	WT	-	3330.7	309.6	0.211	27.3
199	Del(17p)/ p53 M	C	Del/M	F	12403	3.4	0.169	30.6
208	Del(13q)	A	WT	F	24252.8	416.2	0	25.6

To further determine whether sensitivity was a result of defective HRR, cells exposed to 1000 nM talazoparib or vehicle were harvested and HRR function determined by measuring γH2AX and RAD51 foci. A ≥2-fold increase in γH2AX foci was taken as confirmation that stalled/collapsed replication forks and/or DSB had been generated. Cells were defined as HRR competent if a ≥2-fold increase in RAD51 foci was also observed as previously described [20, 21]. Samples 193 and 199 generated analysable results. Both CLL samples were HRR competent (Figure 5B and 5C). Patient 208 also displayed an increase in RAD51 foci indicating HRR competence, however the staining was too poor to analyse fully (data not shown). This suggests that even HRR competent CLL cells are sensitive to talazoparib.

## DISCUSSION

Here we show, for the first time, a comprehensive analysis of the activity and expression of PARP1 in a large cohort of CLL tumours. PARP activity was found to be highly variable among the 109 cases analysed ranging from 200-190,000 pmol PAR/10^6^ CLL cells, i.e. almost 1,000-fold variation. B-cells are reported to have approximately 2-fold higher PARP1 protein levels compared to T cells [22], but some patients had values > 10,000 pmol/10^6^ cells, far higher than previously reported for normal PBMCs. By comparison, PARP activity was less variable in PBMCs from HVs, and although this was from a small cohort (*n* = 8) it was within the range seen in a separate HV cohort (*n* = 56) in our previous study (10 - 2,190 pmol PAR/10^6^cells; [23]). In the CLL cohort studied here, although approximately 1/3 patients had higher PARP activity than HV, median PARP activity was not significantly different in CLL cases, largely due to the huge variation in activity in the cohort.

To understand the variation in PARP activity observed, we measured PARP1 protein levels and discovered that, although there was a significant correlation between protein levels and activity, the strength of the correlation was weak as observed previously [23]. This suggests that the activity of PARP1 is influenced by post-translational modification. To understand this modulation of PARP activity we examined associated clinical data. Treatment status (treatment-naïve as opposed to having received treatment), *IGHV* status, adverse cytogenetic abnormalities or Binet stage did not have a significant impact on the level of PARP activity. However, we noted that there was a trend for PARP activity to be higher in patients with Binet stage B or C and in patients with p53 defects (Figure 3). We therefore examined PARP activity in sequential samples taken from the same patient (Figure S2), but there was no particular trend in PARP activity over the course of disease.

We had hypothesised that PARP activity may be related to underlying genomic instability, (due to p53 or ATM loss), because such cells may be reliant on PARP function for repair of DNA damage. In fact, our data suggest that neither p53 nor ATM loss closely correlate with PARP function. In our cohort, 16/102 patients lacked p53 function, but neither PARP activity nor endogenous PAR levels were significantly related to p53 status. This indicates that in CLL PARP activity is not required in response to the genomic instability and compromised DNA damage signalling resulting from loss of p53. This contrasts somewhat with a recent publication [24] that found *PARP1* expression was lower in CLL samples with mutated *TP53* and unmutated *IGHV*, and that apoptosis positively correlated with *PARP1* expression. However, in that study PARP1 protein levels and activity were only determined in a small subset of samples after irradiation and, because PARP activity is only partially dependent on its protein level and concordance between mRNA and protein levels is highly variable [24, 26], we believe determination of PARP function is a more robust indicator of the cellular phenotype.

Defects in HRR are known to confer sensitivity to PARP inhibitors due to the accumulation of SSB in PARP-inhibited cells that would normally be resolved at replication by HRR [27]. ATM promotes HRR, and lack of ATM has been shown to result in sensitivity to the PARP inhibitor, olaparib in replicating CLL cells [12]. We postulated that CLL cells lacking ATM might be more dependent on PARP and therefore have increased PARP activity. Since del(11q) status alone does not necessarily confer ATM loss (as mutation or loss of the second allele is needed for complete inactivation) we assessed ATM function in selected cases. In 12/46 cases with non-functional ATM, PARP activity was similar to cases with functional ATM, suggesting that ATM dysfunction does not result in an increased reliance on PARP activity. This confirms a lack of association between HRR function and PARP activity previously observed in primary cultures of cells in ovarian cancer ascites [A Mukhopadhyay, PhD thesis, Newcastle University 2011].

To probe the role of ATM and HRR status further, and assess PARP as a therapeutic target in CLL, we analysed the effect the highly potent PARP inhibitor, talazoparib (cell-free IC_50_ = 0.57 nM, [27] on the proliferation of CLL cells using the CD40L-stimulated co-culture model. Here, we used two different culture conditions to allow us to test different exposure times to study the effect of talazoparib on proliferating CLL cells, including the conditions that were previously used [12] and had enough material available to examine a sample which was confirmed as ATM non-functional (CLL 200, Figure 5 and supplementary figure S3) and another with confirmed p53 loss (CLL 199). We found that sensitivity to talazoparib was independent of the ATM functional status of the cells, in contrast to previous studies showing that ATM dysfunctional CLL cells were more sensitive to the PARP inhibitor, olaparib [12]. Duration of exposure to talazoparib at concentrations of 10 nM appears to be important to achieve high levels of cytotoxicity, since samples exposed for 168 hours *versus* 72 hours had lower GI_50_ values. A sub-population of cells appear capable of proliferation even at higher concentrations of talazoparib, and this may reflect an intrinsically resistant sub-population in a heterogeneous population of CLL cells. Our previous studies indicated that a 30 min pulse with talazoparib reduced PARP activity in HV PBMCs cells with an IC_50_ of approximately 7±6 nM (James Murray, unpublished data) suggesting that PARP activity probably needs to be suppressed by > 50% for growth inhibition. In our studies the case with confirmed p53 loss (CLL 199) had similar sensitivity to talazoparib compared to cases with wild type p53. Since p53 loss remains the main indicator of refractory disease, these data present the exciting possibility that talazoparib is effective at reducing proliferation of CLL cells in this difficult-to-treat patient population. All of the samples tested had functional HRR, but this was not associated with resistance to talazoparib. Interestingly, using both method 1 and method 2 to evaluate talazoparib cytotoxicity, the cells with the highest PARP activity, CLL 208 (method 1) and 191 (method 2), were the most sensitive.

Since our data suggested that neither p53 nor ATM status were determinants of intrinsic PARP activity or sensitivity to PARP inhibition, we explored other factors that may alter PARP activity. Bcl-2 is an important pro-survival protein, and a member of a family of proteins that dictate the balance between survival and apoptosis. In CLL, Bcl-2 is frequently highly expressed and associated with disease progression [15] and as such, is being explored as a target in current clinical trials for CLL and other malignancies [28]. We observed high expression of Bcl-2 in the subset of the cohort we analysed, and found that levels of Bcl-2 significantly correlated with PARP expression. Our results suggest that CLL cells may rely more on PARP function, or increase PARP function to promote repair and survival, and in parallel upregulate pro-survival signalling, including Bcl-2 to inhibit apoptosis. In agreement with this, we also found that levels of the NF-κB subunit, RelA, were associated with high PARP activity. NF-κB induces transcription of a large repertoire of stress-response genes in CLL, including the anti-apoptotic proteins Bcl-2 and Mcl-1 [29], allowing us to speculate that stress-induced activation of PARP, and NF-κB in CLL cells occurs as a mechanism of protecting CLL cells from damage.

Endogenous PAR levels varied widely between samples. CLL cells are known to have high levels of reactive oxygen species [11], resulting in increased 8-OHdG DNA lesions that activate PARP. However measurement of 8-OHdG in DNA extracted from a subset of our cohort of CLL samples with high, intermediate or low PAR levels did not reveal a relationship between either endogenous PAR levels or PARP activity and levels of 8-OHdG. It should be recognised that the levels of 8-OHdG represent a balance between induction of DNA damage and its repair, and PAR levels reflect both ongoing synthesis by PARP and degradation by PARG. Therefore, although one might expect high levels of 8-OHdG to be associated with high levels of endogenous PAR, it is possible that in cases where high PAR levels were found in CLL samples with low 8-OHdG, this may have reflected high rates of repair, conversely high levels of 8-OHdG in the absence of high PAR may reflect reduced rates of repair and/or high PARG activity.

We wanted to determine whether PARP activity had any impact on outcome in our cohort. Preliminary analysis using median cut-off values suggested that high PARP activity was associated with poorer overall survival (OS) [13], however, since PARP activity is a continuous variable, we used ROC analysis to more accurately analyse effects on outcome. We found no association with PARP activity and survival, which is in agreement with our analysis of other clinical correlates such as cytogenetic abnormalities and disease progression, where we found that PARP activity was not strongly linked to clinical outcome.

In conclusion, our data suggest that neither PARP1 expression nor activity correlate strongly with adverse cytogenetic abnormalities, p53 or ATM function or stage of disease, but it may be associated with anti-apoptotic proteins. However, encouragingly, our data show that, in an albeit limited number of *ex vivo* cultures, CLL cells were sensitive to talazoparib cytotoxicity independent of their p53, ATM or HRR status, confirming that PARP is a therapeutic target in CLL.

## MATERIALS AND METHODS

### Sample collection and processing

This study was approved by the UK NHS Research Ethics Service, and patient samples are part of the Newcastle Haematology Biobank http://www.ncl.ac.uk/nbb/collections/nhb. Patient information is described in supplementary Table S1. Following written informed consent, patients provided peripheral blood samples which were transported to the laboratory and processed immediately using Lymphoprep (Axis Shield, Cambridgeshire, UK). Sample collection, processing and storage were carried out in accordance with the regulations of the Human Tissue Act 2004 (UK) and local guidelines. Binet staging refers to the patient status at the time of sample collection. All chemicals were obtained from Sigma Aldrich (UK) unless otherwise stated.

### Cytogenetic analyses

Interphase FISH and a multiple ligation dependent probe assay were used to determine cytogenetic abnormalities as previously described [30]. CLL cell DNA was extracted from 5 × 10^6^ lymphocytes using a QIAmp DNA blood mini kit according to manufacturer's instructions (Qiagen, UK). *TP53* mutational status was determined by targeted next generation sequencing (details to be published separately) covering all coding exons (2-11) using a ROCHE 454 GS-FLX platform (performed by NewGene, UK, http://www.newgene.org.uk).

### NF-κB DNA binding activity and expression

NF-κB activity was quantified in nuclear extracts using the Trans-AM NF-кB family Transcription Factor Assay ELISA Kit (Active Motif, Rixensart, Belgium). A standard curve (2 Gy-treated MDA-MB-231 cells) was used to calculate arbitrary units (AU) per μg of nuclear extract from the standard curve.

### Determination of PARP expression and activity

Whole-cell extracts were prepared using Phosphosafe™ Extraction Reagent (Novagen), and proteins separated using Criterion XT 3-8% Tris-acetate (v/v) denaturing polyacrylamide gels (Bio-Rad), transferred onto Hybond C membrane (Amersham) and probed with anti-PARP-1 primary antibody (C2-10, Trevigen, Gaithersburg, MD, USA) then anti-mouse HRP-linked secondary antibodies (Cell Signaling Technology) and ECL reagent (Amersham) detection. Quantification was performed using a Fuji LAS-3000 luminescent image analyzer system (Raytek, Sheffield, UK). PARP activity was determined in 10^3^ digitonin-permeabilised cells (CLL or HV PBMCs) by immunoblotting for the product, PAR, with anti-PAR 10H Ab, following maximal stimulation with a palindromic DS oligonucleotide (5′-CGGAATTCCG-3′; Invitrogen, Paisley, UK 10 μg/ml final concentration) in the presence of excess NAD+ (350 μM) using a GCLP (Good clinical laboratory practise)-validated assay as previously described [23]. CLL cells that had been stored at −80°C were thawed on ice and washed in PBS prior to assay. Endogenous PAR levels were measured in unreacted (i.e. in the absence of oligonucleotide or NAD^+^) cells. Because endogenous PAR levels were low, the maximum number possible were loaded, ranging from 4×10^3^ to 2.5×10^5^ permeabilised cells. Samples were assayed in duplicate, with PARP activity represented as mean recalculated PAR (pmol/10^6^ cells).

### Determination of ATM function

CLL cells were treated with ionizing radiation (IR, 5 Gy, Gulmay Medical Xstrahl RS320 X-irradiator (Gulmay Medical, Chertsey, UK). 45 minutes after IR treatment, cell lysates were prepared as described above and western blots were probed with antibodies to ATM (Abcam, Cambridge, UK), and (where possible) SMC1 (Millipore, MA, USA) and ATM-specific phosphorylation targets; pATM (ser1981) and pSMC1 (ser 966) (Cambridge Bioscience, Cambridge UK). Samples showing clear phosphorylation of ATM (ser1918) and SMC1 (ser966) following IR treatment were deemed ATM functional [4].

### Measurement of Bcl-2 levels

Levels of Bcl-2 were determined in CLL cell lysates, prepared as described above, by Western Blotting using an anti-Bcl-2 antibody (sc-509 Santa Cruz), B-actin (Sigma A4700) and anti-mouse secondary antibody as described above.

### Oxidative DNA damage ELISA

Detection and quantitation of 8-hydroxy-2′-deoxyguanosine (8-OHdG) in DNA was carried out using the 8-oxo-dG ELISA kit II (4380-096-K, Trevigen, Maryland USA). The assay was carried out according to manufacturer's protocol, with the exception of altering the standard curve range from 25 nM - 0.39 nM, to accommodate for lower 8-OHdG levels. 8-OHdG values were interpolated from standard curves, which were generated as per manufacturer's instructions. The final 8-OHdG values represent the amount of 8-OHdG (nM) per μg DNA.

### Growth inhibition by talazoparib of CLL cells cultured on CD40L-expressing cells

CD40L (or non-transfected cells, NTL) cells were seeded into 24-well plates (3×10^5^ cells/ml) in RPMI medium containing 10% heat-inactivated foetal calf serum, 2 mM L-glutamine and 1% penicillin/streptomycin and allowed to adhere. The cells were irradiated (75 Gy) to prevent proliferation and incubated at 37°C overnight. CLL cells were thawed and resuspended at a density of 2 × 10^7^ cells/mL in RPMI supplemented with 10 ng/mL human interleukin-4. Cells were then seeded onto the monolayer at a density of 1×10^7^ per well and incubated at 37°C. Cells were monitored and began to proliferate after approximately 72 hours.

After 72 hours, cells from duplicate wells were counted as a pre-treatment control. In order to determine optimal conditions, experiments were carried out in two ways. Initially (samples 175, 191, 200 and 206), CLL cells were co-cultured with CD40L-expressing cells for 96 hours before being re-seeded onto a fresh feeder layer in culture medium containing talazoparib (provided by BioMarin Pharmaceutical Inc.) for a further 72 hours (‘method 1’). In subsequent experiments (samples 193, 199, 208), 72 hour-cultured CLL cells were transferred onto fresh feeder layers in medium containing talazoparib. Drug-containing medium was replenished with fresh medium containing the same concentration of talazoparib after 72 hours and cells were counted at 24 hour intervals thereafter. Response to drug was measured for the last 72hr (between 96 - 168 hours drug exposure, ‘method 2’). In both instances cell counts (Coulter counter, threshold set at 5-12 μm: Beckman Coulter, High Wycombe, UK) were plotted (a) against time and (b) as a concentration-dependent response curve generated from the cell counts at the terminal 72 hr exposure period as a % of the vehicle control.

### HRR assay

Cells from the CD40L-stimulated cultures that had been treated with vehicle or 1000 nM talazoparib (method 2) were harvested after 144 hours and 1×10^4^ cells/ml cytospun onto microscope slides (Shandon Cytospin 2 centrifuge). Slides were dried, washed 2× in PBS, fixed in cold methanol and stored at −20C. Slides were probed for γH2AX and RAD51 foci as described previously (16) using anti-phospho-Histone H2A.X (Ser139) (clone JBW301, Merck Millipore, Watford, UK), anti-RAD51 antibody (PC130 Calbiochem, Merk Millipore, Watford, UK) and secondary antibodies Alexa Fluor 546 Goat anti-mouse and Alexa Fluor 488 Goat anti-rabbit antibodies (Invitrogen, Life Technologies, Paisley, UK). Coverslips were mounted over the cells in media containing 4′,6-diamidino-2-phenylindole (DAPI) (Vectashield, Peterborough, UK). The number of γH2AX and RAD51 foci was analysed in > 500 cells per condition using ImageJ software with an in-house spot-counting macro [31].

### Statistical analysis

As the data do not have Gaussian distributions, Mann Whitney tests, Kruskal Wallis tests or Spearman rank correlations were performed as indicated in results using GraphPad Prism software (http://www.graphpad.com).

Receiver operating characteristic (ROC) curve survival analysis was performed to assess the predictive accuracy of PARP activity at 2.5 and 5 years (30 and 60 months), [32]. The method uses time-dependent sensitivity, specificity, and associated ROC curves to characterize the predictive accuracy of a continuous marker, when the outcome is a censored survival time. The area under the ROC curve (AUC) was determined using the true positive (TP) and false positive (FP) rate, with an AUC value below 0.75 deemed as having no clinical value. Analysis was carried out in R using the risksetAUC function, part of the risksetROC library R package version 1.0.3, URL: http://CRAN.R-project.org/package = risksetROC). [33]

## SUPPLEMENTARY MATERIAL FIGURES AND TABLE


