# Supplementary material for: PARP1 expression, activity and ex vivo sensitivity to the PARP inhibitor, talazoparib (BMN 673), in chronic lymphocytic leukaemia
Source: Oncotarget. 2015 Nov 2;6(41):43978–91. doi: 10.18632/oncotarget.6287 (PMC4791280; doi:10.18632/oncotarget.6287)
Supplement: Supplementary file 1 [file oncotarget-06-43978-s001.pdf]

PARP1 expression, activity and *ex vivo* sensitivity to the PARP inhibitor, talazoparib (BMN 673), in chronic lymphocytic leukaemia

Supplementary Material

Table S1 Patient Demographics

Details of patients included in the study.

**AGE (years) n = 109**	
Mean	67
Median	67
Range	32-91
**SEX (n = 109)**	
Male	75
Female	34
**BINET STAGE at time of sample collection (n = 103)**	
**A**	50
**B**	18
**C**	35
**ATM FUNCTION STATUS (n = 46)**	
Functional	34
Non-functional	12
***TP53* MUTATION STATUS (n = 102)**	
Wild type	86
Mutant	16
**CYTOGENETIC ABNORMALITIES (n = 108)**	
del(17p)	18
del(11q)	22
Trisomy 12	5
del(13q)	63
***IGHV* MUTATION STATUS (n = 73)**	
Mutated	42
Unmutated	31
**PARP ACTIVITY pmol PAR/10^6 cells (n = 109)**	
Mean	11,967
Median	5,614
Range	192 - 90,052

ENDOGENOUS PAR/10^6 CELLS (n = 89)	
Mean	110
Median	6
Range	0.7 - 3090

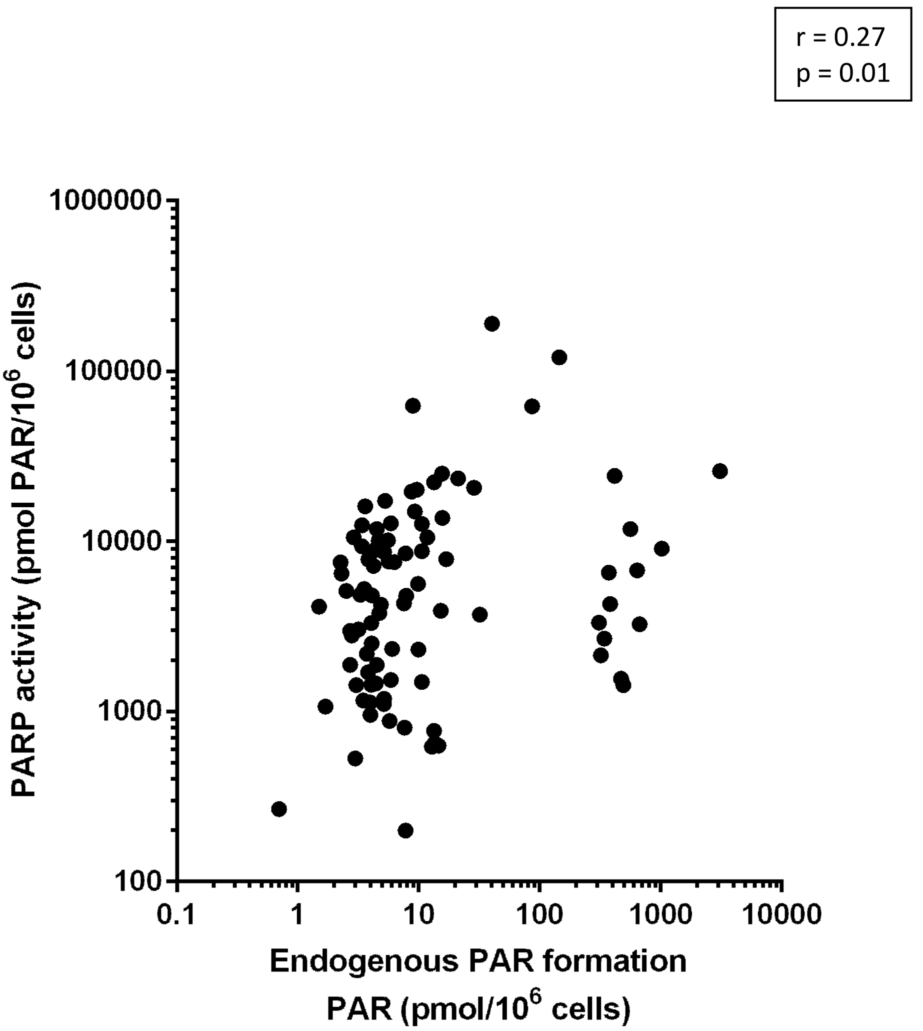

Figure S1. Correlation of Endogenous PAR levels with PARP activity

Paired measurements of PARP activity and endogenous PAR levels were determined in 90 CLL samples. All data are from duplicate determinations in a single assay.

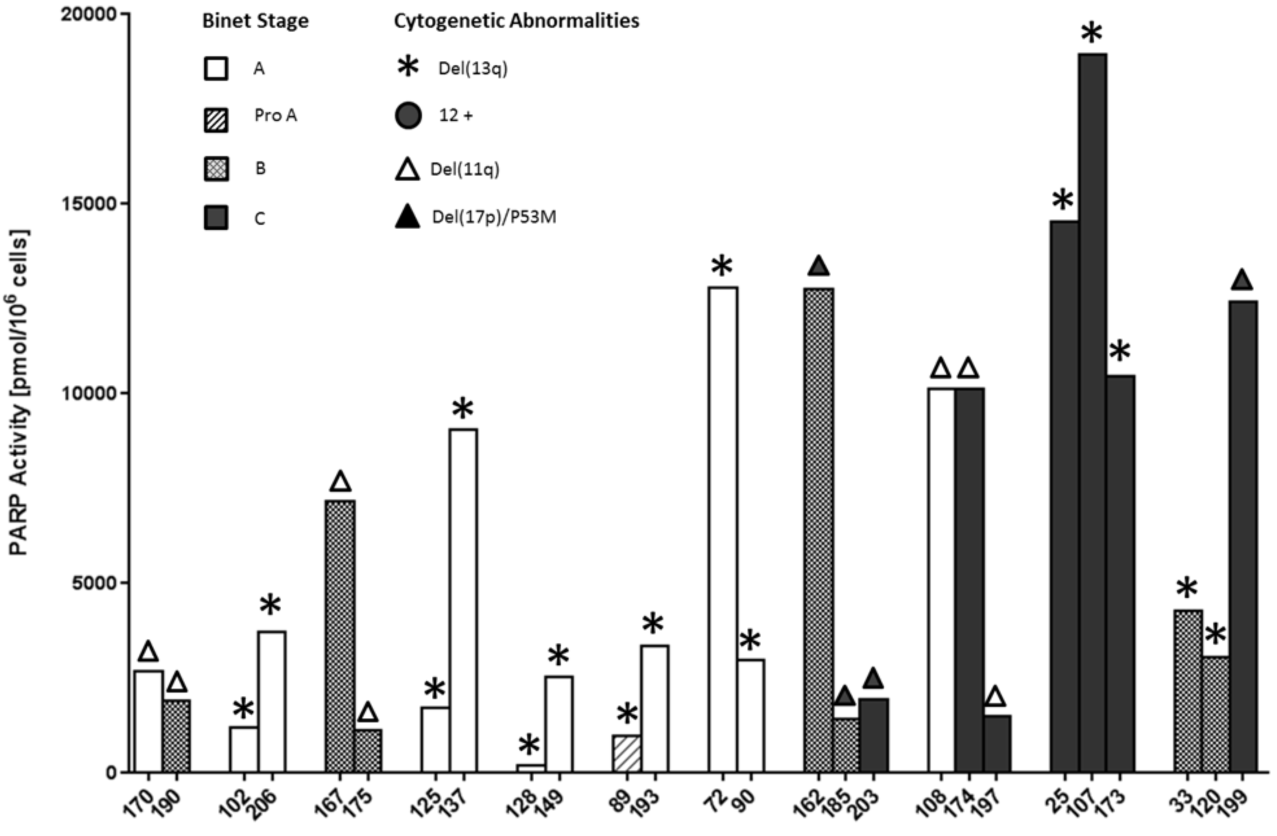

Figure S2. Change in PARP activity with disease course

The data in Figures 1 and 3 included some CLL samples that were taken sequentially from the same patient during disease course (n = 11 patients). To examine PARP activity over the course of disease, PARP activity for the sequential samples from these patients is shown above, with samples from each patient grouped together. These patients include those whose disease had progressed (e.g. 33, 120, 199) and those whose disease remained stable (e.g.102, 206) as judged by cytogenetic abnormalities and Binet stage, which are shown by shading of the bars and symbols above the bars as indicated in the key. All data are from duplicate determinations in a single assay.

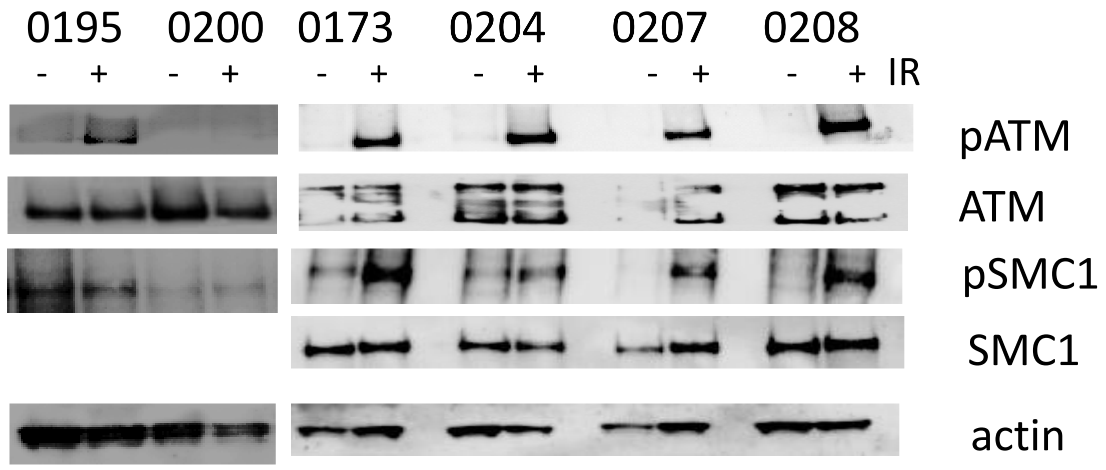

Figure S3. ATM function

ATM function was determined by measuring phosphorylation of ATM-specific targets on ATM itself and SMC1 by Western blotting. Samples were harvested 45 minutes after activation of ATM by 5Gy irradiation ('+' denotes irradiated and '-' denotes mock-irradiated) as described in the Methods. Data are from 2 western blots (SMC1 was not available for samples 0195 and 0200).

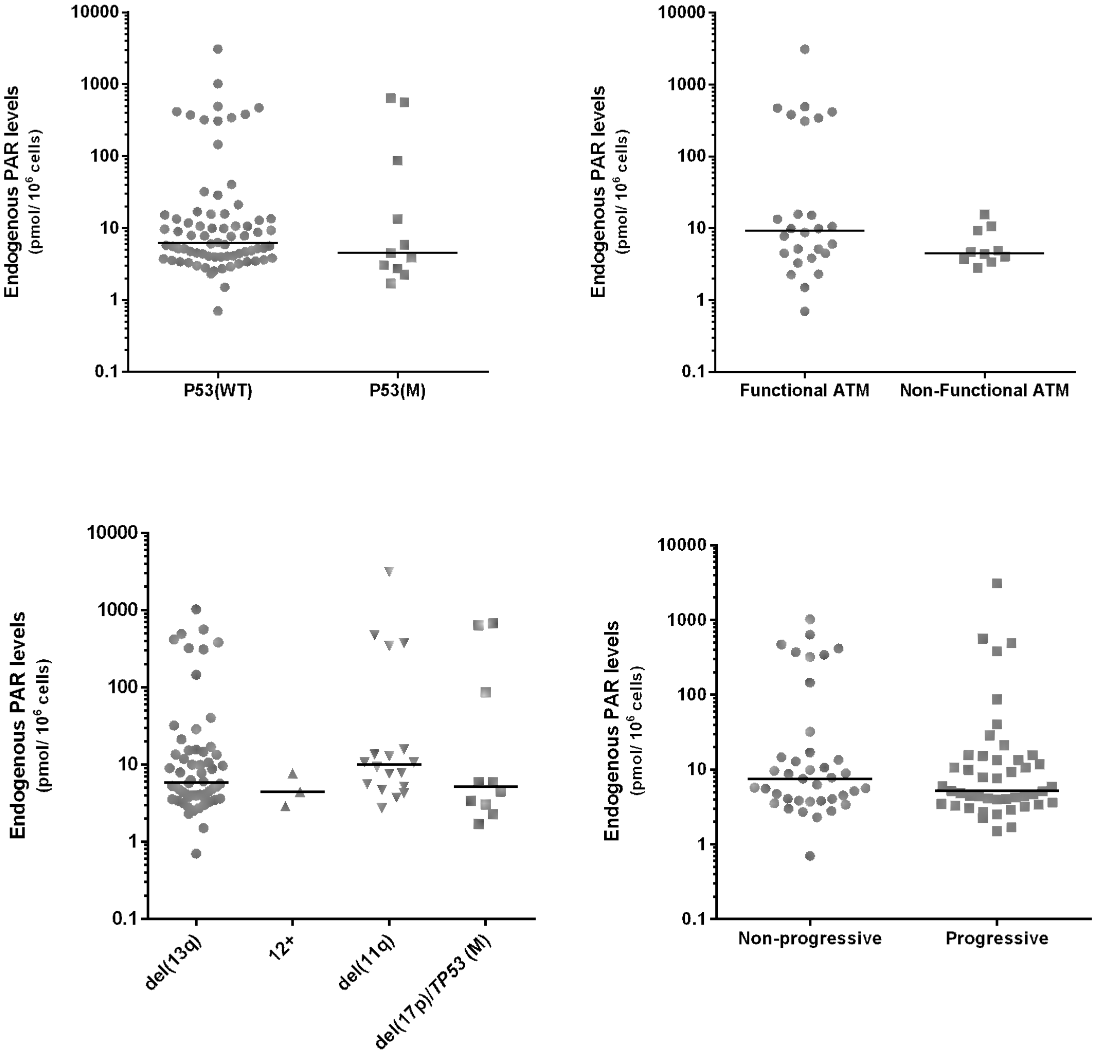

Figure S4. Endogenous PAR levels in relation to p53 and ATM function, cytogenetics and disease status.

Endogenous PAR levels in relation to p53 function (WT n = 86) or mutated (M, n =16) (A), and ATM function (F, ability to auto-phosphorylate (ser1981) and phosphorylate SMC-1, see materials & methods, n=34) or dysfunction (NF n=12) (B). PARP activity in relation to cytogenetic abnormalities (C) and disease status: progressive disease (P, n=53), defined as Binet stage B or C, or non-progressive (NP, n = 50), Binet stage A at the time of sample collection (D). All data are from duplicate determinations in a single assay.

p53/ATM status	PARP activity pmol/10^6 cells mean ±SD (n)	Endogenous PAR pmol/106 cells mean ± SD (n)
p53 WT, ATM F	7466 ± 7662 (n= 28)	287 ± 745 (n= 17)
p53 M, ATM F	16802 ± 8363 (n= 4)	7 ± 5 (n= 4)
p53 WT ATM NF	12428 ± 12233 (n=11)	7 ± 4 (n= 8)
p53 M, ATM NF	12375 ± 4837 (n=3)	N/A (n= 0)

Table S2. PARP activity and endogenous PAR in CLL samples stratified by *TP53* status and ATM function together

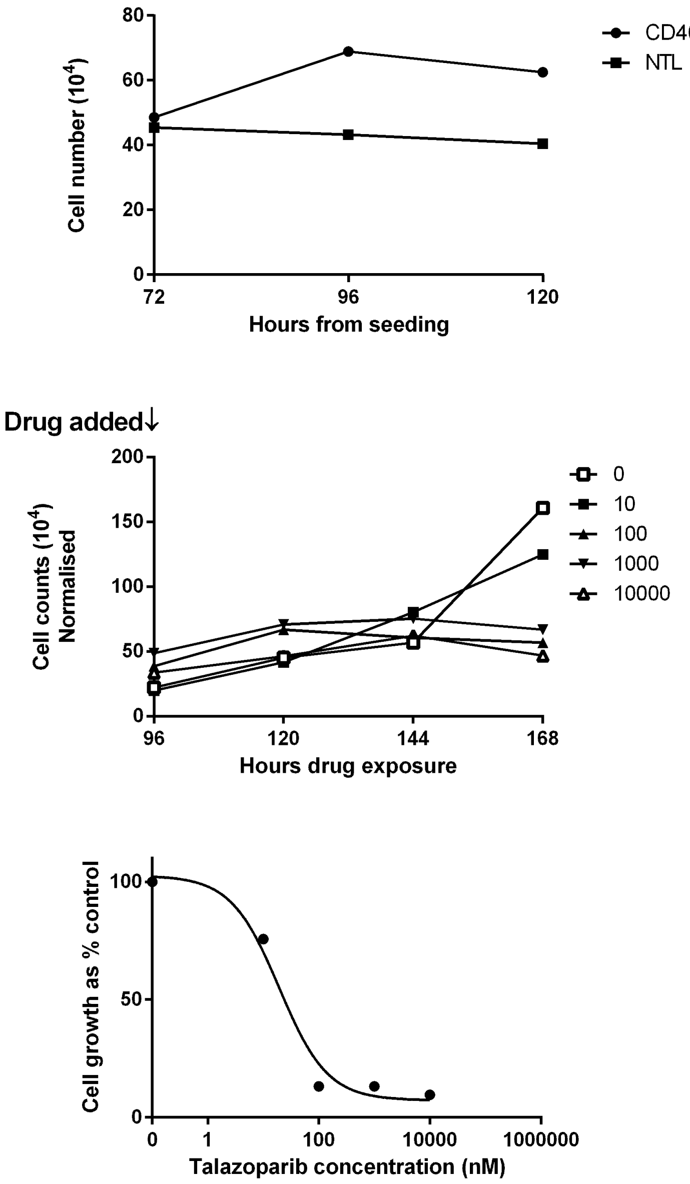

S5. Establishing conditions for CD40L-stimulated CLL cell proliferation (and the effect of talazoparib)

(A) CLL cells were cultured on CD40L and NTL (non-transfected control layer) expressing cells and counted at 72, 96 and 120 hours. CLL cells began to proliferate after approximately 72 hours on the CD40L layer, whereas there was difference in CLL cell number on the NTL layer. (B) CLL cells were cultured on CD40L-expressing cells and treated with increasing concentrations of talazoparib after 72 hours, at the start of proliferation. Cells were counted at 24 hour intervals for a further 72 hours. (C) A concentration-response curve was generated by comparing the CLL cell number after 72 hours of treatment with that at the beginning of drug treatment (CLL sample 191).

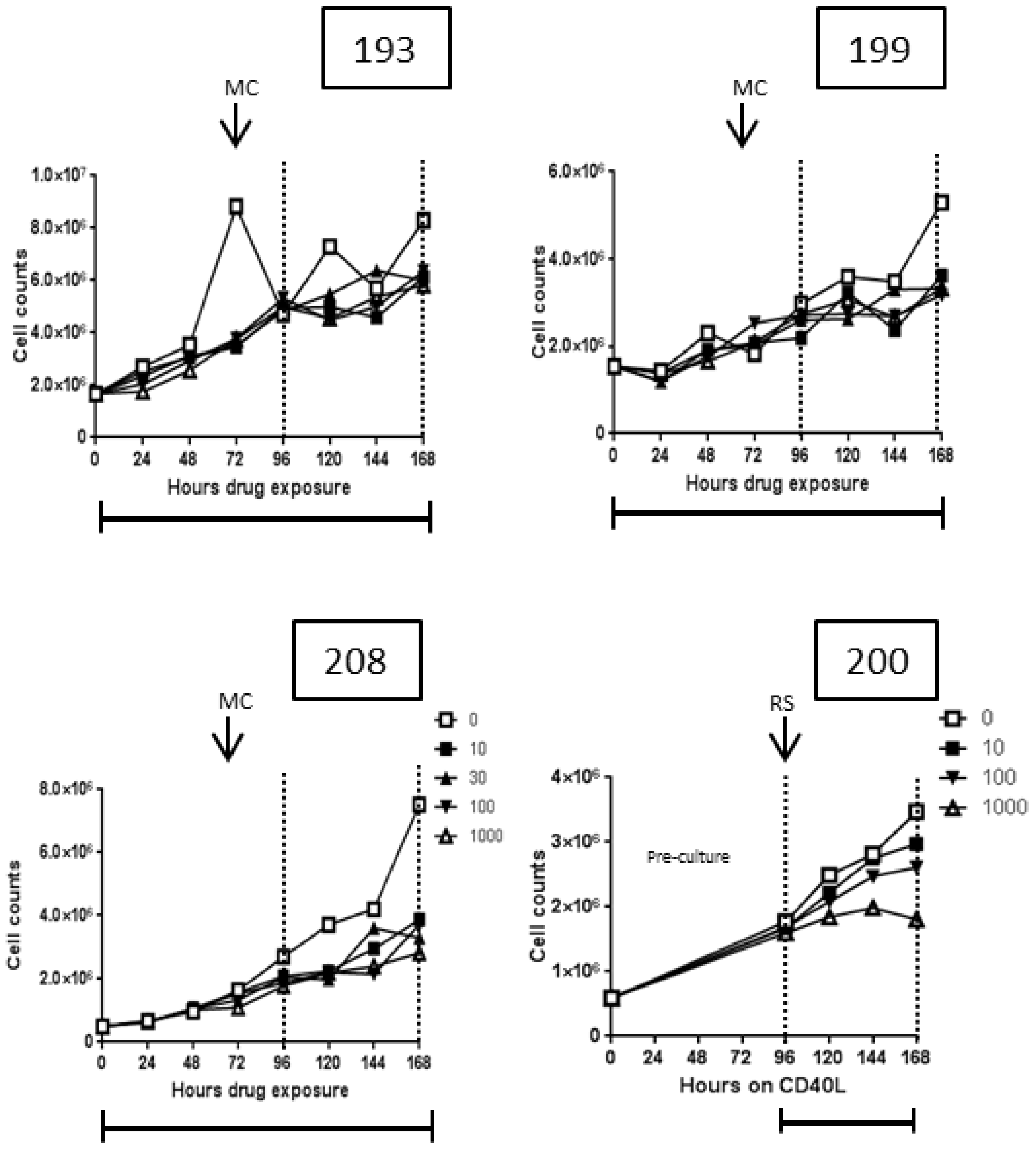

Figure S6. Growth inhibition by talazoparib following different schedules of exposure

Growth of CLL samples following stimulation to proliferate on a CD40L feeder layer and treated with talazoparib (0 nM – 1000 nM) in relation to duration of exposure and concentration of talazoparib after 72 hr exposure during the proliferation phase after refreshment of the media (MC) or re-seeding into drug (RS). The drug exposure periods are marked by a bar. The cell counts taken at day 168 relative to those on day 96 were used to generate the concentration response curves (figure 5).

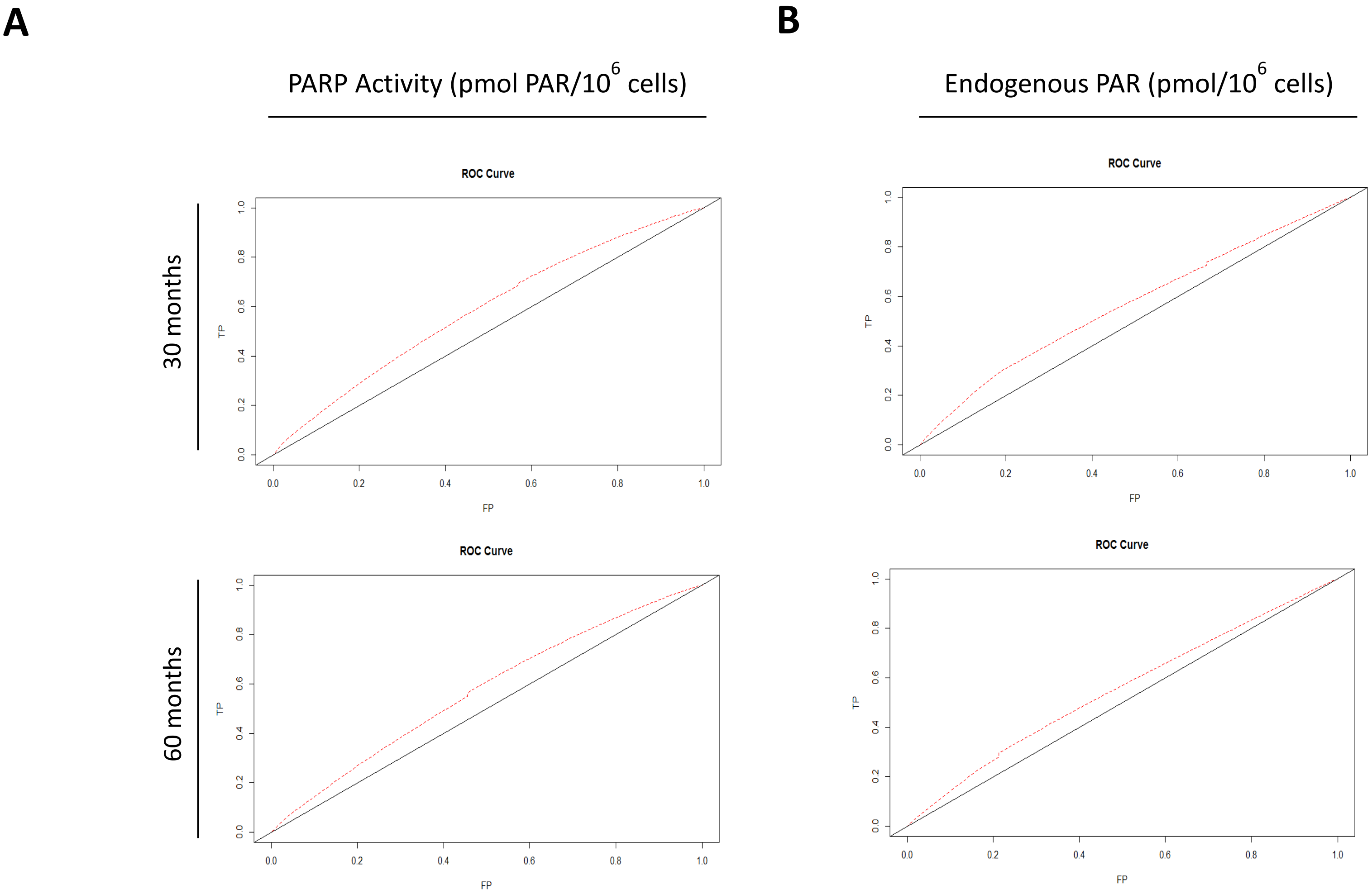

Figure S7. Survival analyses for CLL patients according to PARP activity and endogenous PAR

Receiver operating characteristic (ROC) curves at 2.5 years and 5 years post-diagnosis in relation to PARP activity (A), and endogenous PAR levels (G) showed AUC values of below 0.75, illustrating no clinical significance of PARP as a prognostic marker in CLL.
